# Surgical Outcomes of Craniocervical Junction Fractures in Elderly Patients: A Retrospective Single-Center Series

**DOI:** 10.3390/jpm15100485

**Published:** 2025-10-10

**Authors:** Nicola Montemurro, Stefano Carnesecchi, Riccardo Morganti, Antonella De Carlo, Ardico Cocciaro

**Affiliations:** 1 Department of Neurosurgery, Azienda Ospedaliero Universitaria Pisana (AOUP), 56100 Pisa, Italy; 2 Clinical Trial Statistical Support, Azienda Ospedaliero Universitaria Pisana (AOUP), 56100 Pisa, Italy; 3 Department of Neurosurgery, San Giovanni-Addolorata Hospital, 00184 Roma, Italy

**Keywords:** craniocervical junction, cervical fracture, surgical treatment, elderly, atlas, odontoid, surgical outcome, personalized medicine, axis, precision medicine

## Abstract

**Background:** Fractures of the first and second cervical vertebrae are common in both young and elderly patients. Surgical management of C1–C2 fractures in elderly patients is controversial. The aim of this study is to report the rate of fusion in elderly patients who underwent surgery for C1 or C2 fractures. **Methods:** A retrospective review of all patients over the age of 65 years old who underwent surgical treatment for C1 or C2 fracture was reported. Visual analog scale (VAS) and neck disability index (NDI) were used to assess patients’ clinical outcome at 1 year follow-up. Cervical spine computer tomography (CT) scans were performed in all cases before surgery and at 1 year follow-up to evaluate the long-term postoperative rate of fusion, according to Lenke fusion grade. **Results:** From 2019 to 2023, 105 patients with cervical craniocervical junction (CCJ) fracture underwent surgical treatment in our Pisana University Hospital. Among all these, 74 patients (70.5%) were over 65 years old. The mean age of the study population was 76.9 years old (12.2% aged 65–70, 51.4% aged 70–79, and 36.5% over 80). According to the AO Spine Upper Cervical Injury Classification System, 6 (8.1%) patients presented a type II fracture and 68 (91.9%) patients presented a type III fracture. At admission, neurological examination resulted in American Spinal Injury Association (ASIA) E in 97.3% of cases. Over 60% of all patients underwent C1–C2 posterior fixation. Postoperative complications occurred in 12.25% of patients. According to the criteria described by Lenke, a good rate of fusion (A-B grade) was obtained in 71.6% of patients. **Conclusions:** In elderly patients with CCJ fractures, precision medicine can help identify those at higher risk for complications and guide personalized treatment strategies. Surgical treatment of CCJ fractures in elderly patients, although not always associated with bone fusion, can be performed with an acceptable incidence of mortality and morbidity, allowing rapid mobilization and return to pre-trauma levels of independence.

## 1. Introduction

The atlantoaxial junction is an anatomic complex that involves an intricate relationship between the atlas and the axis to allow flexion, extension, rotation, and lateral bending. Different pathological, trauma, congenital malformation, or inflammatory arthritis have been implicated in the development of atlantoaxial instability with neurological symptoms in the elderly [1,2,3,4,5].

### 1.1. Anatomy of Craniocervical Junction (CCJ)

The second cervical vertebra presents a unique morphology, due to the presence of the odontoid process and very specialized superior facet joints [6,7,8,9]. The complex internal anatomy of C2, its morphology, its morphometric analysis, and its structural properties have been largely reported in the literature [3,10,11,12,13,14,15,16]. Several anatomic studies described the anatomy of the CCJ, which involves a complex arrangement of bony, vascular, and ligamentous anatomy. The C1 ring has two lateral masses that form superior and inferior articular processes. The superior articular process articulate with the occipital condyles, whereas the inferior articular processes articulate with the superior articular processes of C2 [2,3]. The odontoid process is attached to C1 by several ligaments that provide most of the strength of the atlanto-occipital and atlantoaxial structures. The odontoid process is divided into three distinct regions: the tip, the body, and the base [4,5,17]. The structural weakness of the odontoid base coupled with increased bone loss due to age many underlie the high rate of odontoid fractures in the geriatric population.

Atlas fractures represent 3–13% of all cervical spine fractures [18,19,20]. The most well known, eponymously named Jefferson fracture has been defined as a fracture of the atlas from disruption of all types, including unilateral arch fracture, lateral mass fracture, and a combination of C1–C2 fractures [3], whereas type I and II are fractures involving posterior and anterior arch, respectively. Type III is a fracture involving both anterior and posterior arches and type IV is a fracture involving the lateral mass. Atlas fractures can be defined as stable or unstable based on the presumed integrity of the transverse ligament.

The Anderson and D’Alonzo classification [21] is the most commonly used classification of fractures of the odontoid process of C2: type I (fracture of the tip of the odontoid), type II (fracture below the level of the transverse band of the cruciform ligament, potentially unstable with high risk of nonunion), and type III (fracture extending from the base of the odontoid into the vertebral body, relatively stable with the best prognosis for healing) [17,18,19,22,23,24]. According to the Anderson and D’Alonzo classification [21], type II fracture is the most common type (65%) of C2 fractures.

The AO Spine Upper Cervical Injury Classification System [25,26] is based on identifying the upper cervical spine injury location and determining the injury severity based on injury hierarchy. Three anatomically distinct regions of the upper cervical spine were reported as follows: type I (occipital condyle and CCJ), type II (C1 ring and C1–2 joint), and type III (C2 and C2–3 joint).

### 1.2. Cause of CCJ Fractures and Surgical Treatment

C2 fractures occur in both young and geriatric patients, whereas odontoid C2 fractures account for 9% to 15% of adult cervical spine fractures [18]. C1 and C2 fractures are common in the elderly and, due to the aging population, the incidence of these fractures has been increasing. These injuries are usually the result of a hyperextension of the cervical spine due to low-energy impacts in the elderly or high-energy impacts in young and middle-aged people [18,19,22,23]. Neurologic injuries associated with these fractures are rare; in contrast, the rate of nonunion in elderly patients treated conservatively or surgically is common, resulting in high rates of pseudarthrosis [24,27,28]. In addition, these types of lesions are often associated with high morbidity and mortality rates [29,30].

The optimal surgical approach remains unclear. Non-operative treatment options for C1 and C2 fractures include external immobilization with a bivalve collar or halo vest [3,22], whereas surgical treatment includes several anterior or posterior approaches depending on the type of fracture [18,29]. The most common surgical approaches are posterior C1–C2 fusion, according to the Magerl [30] or Goel-Harms [31] techniques, occipitocervical fixation [32], or anterior fusion, such as anterior odontoid screwing [27], and C2–C3 anterior cervical fusion [33].

Geriatric patients with CCJ fractures often have complex comorbidities, making them ideal candidates for precision medicine approaches that tailor care to their unique profiles. Using precision medicine, clinicians can better predict healing capacity and medication response in older adults with cervical spine fractures. The primary objectives of this study were to evaluate the fusion rate and the surgical outcome in this study population older than 65 years old, who underwent surgical fixation for C1 or C2 fractures. Secondary objectives were the assessment of postoperative complications and mortality.

## 2. Materials and Methods

### 2.1. Patient Population

A retrospective review of all patients older than 65 years old, who underwent surgery at our Pisana University Hospital for CCJ post-traumatic fractures, as results of cervical spine injury, was performed between January 2019 and January 2023. Exclusion criteria were patients with cervical pathological fractures and patients with penetrating trauma to the cervical spine. We reviewed sex, age, and the type of fracture according with the new AO Spine Upper Cervical Injury Classification System [25,26,34,35,36], if a post-traumatic subarachnoid hemorrhage (SAH) and/or a non-surgical intracranial hematoma at preoperative CT head scan was present in addition to the cervical fracture, and the American Spinal Injury Association (ASIA) scale at the admission and days of hospital stay [37].

### 2.2. Surgical Outcome

To better understand the surgical outcome and the fusion rate of this study population, we retrospectively collected the type of surgical approach (anterior vs. posterior approach), the type of surgical technique used for fracture fixation (posterior C1–C2 fusion, according to Magerl [30] or Goel-Harms [31] techniques, occipitocervical fixation [32], anterior odontoid screwing [27], C2–C3 anterior cervical fusion [33]). The choice of an anterior or posterior approach and the surgical technique used were decided by the surgeon who performed the surgery, according to the AO Spine guidelines and considering the type of fracture, dislocation, anatomical variants, and comorbidities.

The visual analog scale (VAS) [38] and the neck disability index (NDI) as % (0–100) [39] scores were used to evaluate surgical outcomes at 1 year follow-up. Similarly to previous papers [40,41,42] that classified the fusion rate using the Bridwell/Lenke fusion classification [43], cervical fusion grade was assessed on the criteria described by Lenke et al. [44] (Table 1). In addition, any hardware failures were reported. Additionally, changes in alignment and signs of myelopathy were assessed.

Medical records were reviewed retrospectively from the time of surgery until the patient’s discharge to assess postoperative surgical complications. Mortality at 3 months and 1 year was assessed by phone follow-up.

### 2.3. Statistical Analysis

Categorical data were described with frequency and percentage; continuous data were summarized with mean and standard deviation. To compare categorical factors with categorical outcomes, Chi square test was applied. To analyze the categorical factors influencing continuous outcomes, t-test for independent samples was used. Successively, multiple linear regression was performed as multivariate analysis. Significance was set at 0.05 and all analyses were carried out by SPSS version 29.

## 3. Results

**Figure 1:**
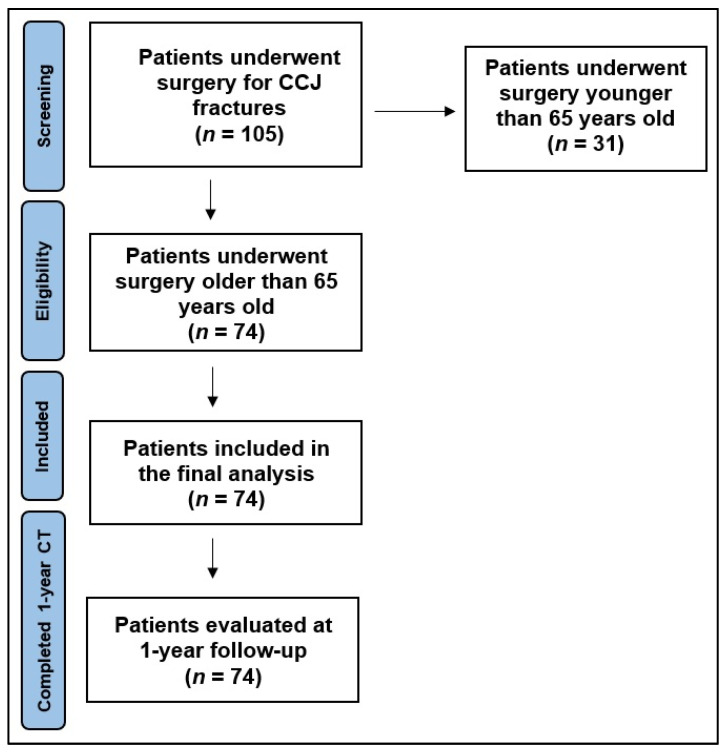
Flow diagram of patient selection into the study cohort.

Between January 2019 and January 2023, 105 patients with CCJ fractures underwent surgical treatment in our Pisana University Hospital. Among all these, 74 patients (70.5%) were over 65 years old, with 33 (44.6%) male and 41 (55.4%) female (Figure 1). The mean age of the study population was 76.9 years old (12.2% aged 65–70, 51.4% aged 70–79, and 36.5% over 80 years old). According to the AO Spine Upper Cervical Injury Classification System [25,26,34,35,36], 6 (8.1%) patients presented a type II fracture and 68 (91.9%) patients presented a type III fracture. At admission, neurological examination of these patients resulted in ASIA E in 97.3% of cases. In 20 patients (27%), the cervical C1 or C2 fracture was associated with a positive CT head scan for a post-traumatic SAH and/or a non-surgical intracranial hematoma (IH). The average overall hospitalization was 10.6 days (range 7–30 days). The mean clinical follow-up was 15 months. Table 2 shows all details.

**Figure 2:**
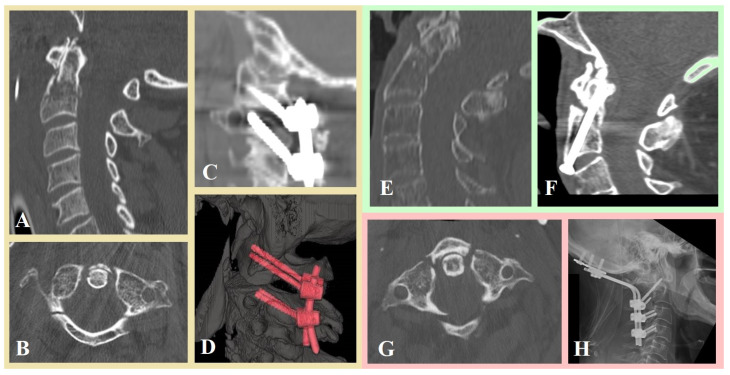
Preoperative cervical CT scans (**A**,**B**) of an 81-year-old man with C1 and C2 fractures underwent C1–C2 fixation according to Harms technique (**C**,**D**). Preoperative cervical CT scan (**E**) of an 83-year-old man with an odontoid C2 fracture type II according to Anderson and D’Alonzo classification underwent C2 dens screwing (**F**). Preoperative cervical CT scan (**G**) of an 82-year-old woman with a Jefferson type III C1 fracture treated surgically with occipitocervical fixation (**H**).

Thirty (40.5%) patients underwent fixation through an anterior approach, whereas 44 (59.5%) patients underwent a posterior approach. In detail, 22 (29.7%) patients underwent C2 dens screwing and 8 (10.8%) patients underwent C2–C3 discectomy, to replace disc with cage and fixation with plate, whereas 11 (14.9%), 25 (33.8%) and 8 (10.8%) patients underwent occipitocervical fixation, C1–C2 fixation according to Harms technique [31] and C1–C2 fixation according to Magerl technique [30], respectively. Figure 2 shows three clinical cases from our series.

Postoperative complications occurred in 12.2% of patients, with a 3-months mortality rate of 4.1% and 1-year mortality rate of 17.6%. Five (6.8%) patients experienced wound infection, whereas one patient had postoperative hematoma in the surgical site, which required surgical evacuation as an emergency, and three (4.1%) patients developed deep vein thrombosis, despite a relatively rapid mobilization. Overall surgical outcome was good, as mean VAS and mean NDI were 2.95 and 5.9%, respectively, at 1 year follow-up. Among all 74 patients, only 71.6% of patients received a good fusion (grade A-B according to Lenke et al. [44]) at 1 year follow-up, with a fracture nonunion rate of 28.4% (grade C–D according to Lenke et al. [44]). No hardware failures, neither screw pullout, were reported. Table 3 shows all details.

### Statistical Analysis on Clinical and Fusion Rate Outcome

A retrospective, deep analysis of prognostic factors that affected outcome, mortality, and fusion rate was conducted. Age, type of fracture, and surgical choice of treatment does not affect clinical outcome and surgical complications, even if type II C seems to be more related with a higher mortality at 1 year follow-up. Although not statistically significant, a trend (*p* = 0.067) showed that a posterior approach, regardless of the technique used (occipitocervical fixation, or C1–C2 fixation according to Harms or Magerl technique), was related to a higher fusion rate compared to the reported anterior approach (C2 dens screwing and C2–C3 discectomy and fixation with plate) in this study for CCJ fractures (Table 4).

A univariate and a multivariate analysis were performed to assess which factors affect long-term cervical pain after CCJ fractures in older patients at 1 year follow-up. From this analysis (Table 5), it resulted that patients older than 80 years old had a higher NDI compared to younger patients. Similarly, patients with type II type C fractures had a higher NDI compared to other kinds of fractures. A posterior surgical approach for CCJ fractures was related to a higher VAS score (*p* < 0.001) at 1 year follow-up, compared to all the anterior surgical approaches.

## 4. Discussion

The choice of surgical approach seems to be particularly important in elderly patients, as well as in so-called frail patients, because this may correlate with a better or worse quality of life and return to normal daily activities. We reported that posterior surgical approaches for CCJ fractures were related to a higher VAS score at 1 year follow-up, and, in addition, they seem to be associated with a higher fusion rate compared to anterior approaches, although not statistically significant. Several surgical techniques have been introduced for atlantoaxial fusion (C1–C2), the most common being Magerl’s (transarticular) or Harms/Goel screw fixation or posterior extension of fixation to C3–C4 or the occiput [31,45,46]. More challenging techniques have also been proposed, such as transoral atlantoaxial reduction with plate, anterior transarticular screw fixation, and direct transpedicular C2 fixation (Judet approach) [47,48,49].

Both anterior and posterior approach provide immediate stability to the fracture [10,32,50]. Some disadvantages of the anterior screw include dysphagia, respiratory-related complications, and in elderly patients, osteoporosis can predispose to screw failure [16,51]. Contraindications to anterior screw fixation include fracture comminution, oblique anterior fractures, cervical spondylosis, and kyphosis and short necks. Posterior approach consists of transarticular screws positioning at C1–C2, lateral mass screws in C1 with pedicle, or interlaminar screws at C2. C1–C2 fusion results in the loss of approximately 50% of axial rotation in the cervical spine; this is less tolerated in geriatric patients than younger patients [10,31,52].

### 4.1. Functional Outcomes

The best management of temporomandibular joint (CCJ) fractures in older adults remains controversial. To improve surgical and clinical outcome, a personalized approach should be considered [32,36], as many concomitant diseases such as temporo-mandibular disorders, fibromyalgia, rheumatoid arthritis can influence clinical outcomes [53]. The best management for CCJ fractures in the elderly remains controversial. In order to improve surgical and clinical outcome, a personalized approach must be considered [18,24]. Posterior approach seems to give longer time to recover from pain than anterior approach [18,24]. The NDI was higher in elder patients (>80 yrs) [24]. Functional recovery after surgical intervention for CCJ fractures in elderly patients remains a critical concern given the high risks of morbidity and mortality associated with this population. Several studies indicate that despite the complexity of these fractures and the frailty of patients, a significant proportion of survivors can return to their pre-injury level of independence, though with variable rates of functional decline [54]. Sander et al. [54] reported 69 elderly patients undergoing surgery for cervical spine fractures and found that approximately 82% of patients who lived independently before injury returned to their pre-injury residence after treatment, suggesting that surgery can help maintain functional independence in many cases. In the same study [54], surgical treatment for cervical spine fractures showed a 3-month mortality of 26.1% and in-hospital mortality of 21.7%. Mortality was significantly associated with older age and the presence of neurological deficits.

Rizvi et al. [28] reported in their study with 282 patients with Andreson type II fracture that primary conservative treatment appears to be the best approach in respect to the proportion of pseudarthrosis and neck pain in elderly patients. On the other hand, the De Bonis et al. [55] series reported that 79,8% of patients showed good outcome with anterior odontoid fixation, while C1–C2 posterior arthrodesis and occipitocervical stabilization were associated with worse NDI.

Sasagawa et al. [56], focusing on fusion surgery in elderly patients, reported a 33% rate of deterioration in walking ability at follow-up, underscoring the importance of not only achieving spinal stability but also the need for comprehensive rehabilitation and close monitoring of neurological function postoperatively. Factors such as higher comorbidity scores (Charlson Comorbidity Index), more severe neurological deficits at admission, and longer surgical times were associated with poorer functional outcomes [56].

In our series, postoperative complications (12.2%) were not related to age, type of fracture, and type of surgical treatment. Elderly patients frequently had cardiac, pulmonary, renal, or endocrine comorbidities at diagnosis. Harrop et al. [57] recommended, in the elderly (>65 years), surgery for Anderson II fractures and conservative treatment in Anderson III fractures. Other authors [5,28,58] confirm that a conservative approach to odontoid type II fractures in the elderly (>75 years) is an effective and valid option, resulting in an excellent functional outcome in the majority of cases.

In recent years, cervical fixation precision medicine has involved using personalized approaches, such as 3D printing for custom orthoses and advanced surgical techniques, to improve the stability, comfort, and outcomes of cervical spine treatments for individual patients [59,60].

### 4.2. Mortality

While surgery generally improves fusion outcomes, it is accompanied by increased perioperative risks. The decision for surgery should carefully balance the higher fusion rates against the increased risk of complications and longer hospital stays.

Delcourt et al. [61], in their review of upper cervical fractures in the elderly, reported a wide variability in outcomes: mortality ranged between 0% and 31.4%, and morbidity between 10.3% and 90.9%. At the same time, no definitive superiority of any treatment modality (collar, halo, or surgery) was demonstrated. Chen et al. [62], comparing surgical vs. nonoperative treatment for closed C2 fractures in patients older than 65 years old, found no statistically significant differences at 30-day mortality (3.6% vs. 7.1%) or complication rates (17.9% vs. 25.0%) and in long-term survival, suggesting age alone is not a contraindication to surgery. Chan [63], focusing on C2 spinal fractures in patients older than 65 years old, reported mortality rates of 14.3% and 35.7% at day 30 and 1 year, respectively. Factors such as higher CCI, more severe neurologic impairment per the ASIA scale, longer surgery, low albumin, low hemoglobin, and surgeries involving more levels were linked to increased mortality and functional decline. The integration of intraoperative neurophysiological monitoring (IONM) in the surgical management of vertebral fractures has contributed to improved safety and outcomes in this challenging patient population [64].

### 4.3. Fusion Rates

Achieving successful spinal fusion is a primary surgical goal in managing CCJ fractures, especially in C2 fractures, which are known for their propensity for nonunion due to limited vascularity and motion at the fracture site. A systematic review and meta-analysis [6], focusing on elderly patients with type II odontoid fractures, reported significantly higher fusion rates (74.3% vs. 40.3%), and lower mortality (13.2% vs. 19.0%), despite elevated complication rates (26.0% vs. 18.5%) and longer hospitalization (13.6 vs. 8.1 days) in surgery compared to conservative management. This improved fusion was associated with better stability and potential reductions in late complications such as chronic pain and deformity. In our study, a posterior approach gives better results in terms of bone fusion rate than any kind of anterior approaches. Dobran et al. [65] observed that in 20 patients, posterior fixation with polyaxial C1 lateral mass screws and C2 pars screws produced a solid fusion with atlantoaxial fixation and it is a safe and effective option in the treatment of odontoid fractures with long-term stability and less vascular or neurological complication.

Preoperative evaluation of osteoporosis must be considered, especially in the elderly or in patients with short necks and high body mass index; cement augmentation may be an option to reduce the postoperative risk of screw loosening [51]. Gembruch et al. [51] showed 30.8% progressive loosening of the screws in their patients treated with anterior transarticular C1–C2 fixation. Navigation with 3D reconstruction reduces complications, radiation exposure, and surgical time [66]. Exoscope seems to be a safe alternative compared to an operative microscope, when necessary, with several advantages that have been achieved, such as easier simplicity of use and better 3D vision and magnification of the surgical field and offers the opportunity of better interaction with other members of the surgical staff [67,68].

### 4.4. Study Strengths and Limitations

The current study is a retrospective analysis of non-randomized consecutive patients that underwent surgery for C1–C2 cervical fractures. First, it is difficult to define the forces acted on the cervical spine and it remains difficult to quantify the exact energy impact applied to the cervical spine during trauma in each patient. Secondly, our study reflects a single-center experience with a relatively small number of patients treated at the same hospital. Third, due to the retrospective nature of the study, some data were missing, like some minor postoperative complications (like urinary tract infections) or comorbidity (osteoporosis grade, chronic renal failure, hematopoietic disease, and rheumatological disease), that can affect overall clinical outcome and fusion rate. Lastly, absence of standardized indications, heterogeneity of procedures, and potential selection bias may have altered the reported results and statistical analysis. Additional prospective studies should be conducted in an international, multi-center setting, probably with a longer follow-up period.

## 5. Conclusions

Surgical intervention for C1 or C2 cervical fractures in elderly patients can offer survival benefits and better fracture healing, particularly for displaced fractures. However, the increased risk of complications and mortality in patients over 80 years necessitates a careful, individualized approach. Conservative management may be appropriate for patients with minimal displacement and good overall health. Surgical stabilization of CCJ fractures in the elderly improves fusion rates significantly, which is crucial for mechanical stability and preventing late complications. Functional outcomes can be favorable, with a large number of patients maintaining independence; however, postoperative neurological decline and walking impairments remain challenges. Therefore, emphasizing the importance of analysis in clinical practice is crucial in bridging the gap between theory and practical application, ensuring that surgical advancements lead to tangible benefits for patients. The risks of surgery in the elderly population must be carefully evaluated on a case-by-case basis. Precision medicine can support the development of personalized rehabilitation programs following CCJ injuries in older adults. By applying precision medicine principles, healthcare providers can improve functional outcomes and reduce mortality in older patients with cervical spine fractures. These findings underscore the need for personalized treatment plans and rigorous postoperative care to optimize both survival and quality of life.

## Figures and Tables

**Table 1:** Lenke et al. [44] classification for posterolateral fusion grade/score.

Fusion Grade	Description of Fusion
A	Solid, big trabeculated fusions bilaterally
B	Solid unilateral fusion with small contralateral aspect
C	Small thin fusion masses bilaterally with possible crack
D	Graft resorption bilaterally or bilateral pseudoarthrosis

**Table 2:** Demographic population study.

	*n*° (%)
Patients	74 (100)
Age (mean)	76.9
Sex	
Male	33 (44.6)
Female	41 (55.4)
Type of fracture	
type II	6 (8.1)
type III	68 (91.1)
ASIA scale	
Asia C	2 (2.7)
Asia E	72 (97.3)
Positive CT head scan for SAH or IH	20 (27)
Stay in hospital (mean, days)	10.6
Follow-up (mean, months)	15

SAH, subarachnoid hemorrhage; IH, intracranial hematoma.

**Table 3:** Surgical treatment and outcome.

	*n*° (%)
Surgical treatment	74 (100)
C2 dens screwing	22 (29.7)
C2–C3 discectomy and fixation with plate	8 (10.8)
Occipitocervical fixation	11 (14.9)
C1–C2 fixation Harms technique	25 (33.8)
C1–C2 fixation Magerl technique	8 (10.8)
Postoperative complications	9 (12.2)
wound infection	5 (6.8)
postoperative hematoma	1 (1.4)
vein thrombosis	3 (4.1)
Clinical outcome	
VAS (mean)	2.95
NDI (mean)	5.9%
Radiological outcome (Lenke et al. [44])	
Fusion grade A–B	53 (71.6)
Fusion grade C–D	21 (28.4)
Mortality	
at 3 months	3 (4.1)
at 1 year	13 (17.6)

VAS, visual analog scale; NDI, neck disability index.

**Table 4:** Statistical analysis of prognostic factors on outcome.

	Postoperative Complications			Mortality at 1-Year FU			Fusion Rate		
Prognostic Factors	No	Yes	*p*-Value	No	Yes	*p*-Value	Fusion Grade C–D	Fusion Grade A–B	*p*-Value
Gender			0.969			0.624			0.850
Male	29	4		28	5		9	24	
Female	36	5		33	8		12	29	
Age			0.257			0.496			0.285
<80 y.o.	42	4		39	7		11	35	
≥80 y.o.	23	5		22	6		10	18	
Type of fractures (yes)									
Type II, type B	3	1	0.428	4	0	0.342	2	2	0.324
Type II, type C	2	0	0.706	0	2	0.002	0	2	0.367
Type III, type B	43	4	0.218	41	6	0.190	10	36	0.104
Type III, type C	17	4	0.267	16	5	0.374	9	12	0.082
Surgical treatment (yes)									
Occipitocervical fixation	9	2	0.522	8	3	0.359	2	9	0.416
C2 dens screwing	19	3	0.823	18	4	0.928	9	13	0.120
C1–C2 fixation Harms	22	3	0.975	20	5	0.694	5	20	0.253
C1–C2 fixation Magerl	7	1	0.988	7	1	0.690	2	6	0.822
C2–C3 anterior fixation	8	0	0.261	8	0	0.167	3	5	0.545
Surgical approach			0.613			0.429			0.067
Posterior	38	6		35	9		9	35	
Anterior	27	3		26	4		12	18	

**Table 5:** Statistical analysis of prognostic factors on long-term cervical pain at 1 year follow-up.

	VAS					NDI				
	Univariate Analysis			Multivariate Analysis *		Univariate Analysis			Multivariate Analysis *	
Prognostic Factors	Mean	sd	*p*-Value	RC	*p*-Value	Mean	sd	*p*-Value	RC	*p*-Value
Age										
<80	2.85	1.32	0.381			4.93	3.85	0.013	3.08 (1.17; 4.98)	0.002
≥80	3.11	1.07				7.54	4.90			
Gender										
Male	2.94	1.37	0.967			6.12	4.04	0.727		
Female	2.95	1.12				5.76	4.76			
Type of fracture (yes)										
Type II, type B	4.25	1.26	0.028	0.66 (−0.36; 1.67)	0.199	5.00	1.63	0.673		
Type II, type C	3.50	2.12	0.521			14.50	9.19	0.005	8.67 (2.67; 14.7)	< 0.005
Type III, type B	3.04	1.23	0.384			5.72	4.43	0.619		
Type III, type C	2.52	0.93	0.062			5.71	3.91	0.805		
SAH or IH at CT scan										
no	2.98	1.25	0.626			5.38	3.76	0.045	1.67 (−0.67; 4.02)	0.160
yes	2.81	1.17				7.88	6.05			
Surgical treatment (yes)										
Occipitocervical fixation	3.64	0.67	0.042	0.10 (−0.60; 0.80)	0.770	7.27	6.15	0.275		
C2 dens screwing	2.05	0.95	<0.001	*	*	6.68	5.34	0.339		
C1–C2 fixation Harms	3.44	0.96	0.012	*	*	4.96	2.75	0.186		
C1–C2 fixation Magerl	4.00	1.31	0.009	0.50 (−0.27; 1.28)	0.197	6.88	4.85	0.522		
C2–C3 anterior fixation	1.88	0.83	0.004	−0.17 (−0.95; 0.61)	0.664	4.00	1.60	0.098		
Surgical approach										
Posterior	3.59	0.97	<0.001	−1.37 (−1.92; −0.81)	<0.001	5.89	4.24	0.940		
Anterior	2.00	0.91				5.97	4.77			
*Constant*				3.4 (3.03; 3.79)	<0.001				4.16 (2.88; 5.44)	<0.001

* Variable excluded from the model. VAS, visual analog scale; NDI, neck disability index.

## Data Availability

The data are available in the tables in this manuscript. Other data used to support the findings of this study are available from the corresponding author upon request.
